# CD-20 Negative Plasmablastic Lymphoma Lurking in the Shadow of a Leiomyoma – Diagnosis and Management

**DOI:** 10.7759/cureus.5217

**Published:** 2019-07-23

**Authors:** Bikramjit S Bindra, Gowthami Ramineni, Yasar Sattar, Ratesh Khillan

**Affiliations:** 1 Internal Medicine, Government Medical College and Hospital, Chandigarh, IND; 2 Internal Medicine, Rajiv Gandhi Institute of Medical Sciences, Ongole, IND; 3 Internal Medicine, Icahn School of Medicine at Mount Sinai, New York, USA; 4 Hematology / Oncology, Kingsbrook Jewish Medical Center, New York, USA

**Keywords:** plasmablastic lymphoma, cd-20 negative lymphoma, treatment, diagnosis

## Abstract

CD20-negative diffuse large B-cell lymphoma (DLBCL) is a rare entity and constitutes 1-2% of all DLBCLs. Major subtypes include plasmablastic lymphomas (PBLs), primary effusion lymphomas, anaplastic kinase positive large B-cell lymphomas, and large B-cell lymphomas arising in human herpesvirus 8 (HHV8)-associated multicentric Castleman disease. Amongst the known subtypes, PBL is the most common and presents as an aggressive extranodal disease with high resistance to routine chemotherapy regimens, thereby posing a therapeutic challenge. Though more commonly seen in HIV-positive patients, PBL cases have also been reported in HIV negative patients. We report a unique case of PBL with pelvic organ involvement in an HIV/Epstein-Barr virus-negative patient. The neoplastic cells were found to be positive for CD79a, MUM1, BCL6, and PAX5, with a Ki-67 proliferation index of 92%. Our case met the criteria for the plasmablastic variant, and remission was obtained with etoposide, vincristine, and doxorubicin with bolus doses of cyclophosphamide and oral prednisone (EPOCH) therapy. This case report aims to highlight the challenges related to the diagnosis and treatment of CD20-negative DLBCL, with special emphasis on the PBL subtype and to provide an insight into some of the upcoming, less conventional treatment modalities.

## Introduction

Diffuse large B-cell lymphoma (DLBCL), a non-Hodgkin lymphoma (NHL) of B-cell origin, accounts for approximately 30-35% of cases of NHL in general population. Amongst the DLBCLs, CD20-negative subtype represents a rare and heterogeneous group of high-grade aggressive non-Hodgkin’s large B-cell lymphomas [1,2]. The decreased expression of CD20 and PAX5 and increased expression of CD38, CD138, and CD56 are associated with poor B-cell differentiation, high resistance to chemotherapy, and a poor prognosis [3]. These lymphomas share a plasmablastic differentiation profile due to the gradual expression of plasma cell transcription factors like PRDM1/BLIMP1, MUM1/IRF4, and XBP1s [3-5]. Iatrogenic loss of CD20 in cases of DLBCL have been reported status post-rituximab treatment [6]. De novo CD20- DLBCL has been reported in HIV cases [7,8]. Here, we report a case of de novo CD20- DLBCL in an HIV negative patient. We have also outlined the management, diagnosis, and literature search for similar cases.

## Case presentation

A 57-year-old African American woman with a past medical history of hypertension and uterine fibroids was transferred from outpatient gynecology to the emergency department with complaints of fever, shortness of breath, dizziness, lower abdominal/pelvic pain, abnormal uterine bleeding, and pelvic fullness. At presentation, she was febrile with a body temperature of 100°F. The rest of her vitals were stable; her blood pressure was 122/80 mmHg, heart rate was 87 beats/minute, and respiratory rate was 16 breaths/minute. Physical examination was significant for gross pallor. She was alert and oriented to time, place, and person. Her abdomen was diffusely tender without any peritoneal signs and was distended with multiple palpable, fixed, firm masses. Her lower abdomen was dull to percussion. A pelvic examination revealed multiple palpable masses in the uterus with some active bleeding from the cervical os. The rest of the physical examination was normal. Her electrocardiogram (EKG) showed normal sinus rhythm. Her chest radiography was normal. Her laboratory results were significant for a hemoglobin level of 6.6 g/dL, reticulocyte count 2.9%, serum iron 13 mcg/dL, ferritin 1332 ng/ml, serum lactic acid dehydrogenase (LDH) 451 U/L, haptoglobin 371 mg/dl, c-reactive protein (CRP) 22.07 mg/dl, erythrocyte sedimentation rate (ESR) of 115 mm/hr, and prothrombin time and international normalized ratio (INR) of 50.8 seconds and 2.44, respectively. The rest of her laboratory results, including the tumor markers CA 125, alpha-fetoprotein (AFP), and human chorionic gonadotropin (HCG), were within normal limits.

**Figure 1:**
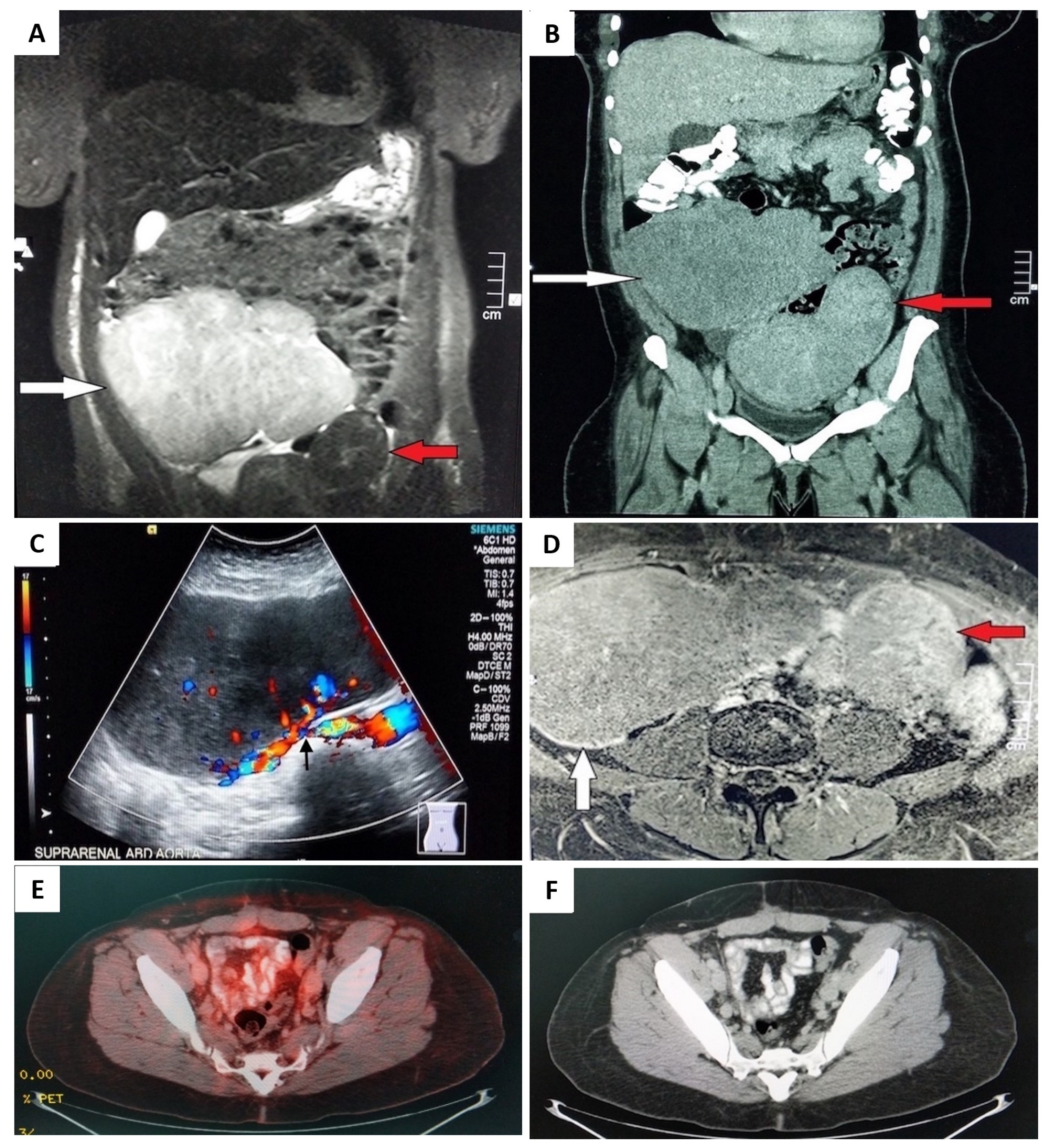
Radiology images at the time of presentation. A coronal T2W MRI (A) and coronal CT (B) images demonstrating an exophytic mass (red arrow) arising from the uterus (leiomyoma) and a large tumor mass (white arrow) with high signal intensity on MRI. A trans-abdominal ultrasound (C) demonstrating a large tumor mass with increased vascularity (arrow). An axial T2W MRI image (D) showing a large tumor mass (white arrow) and left tumor mass (red arrow). Post-therapeutic radiology images (E, F): A PET/CT image (E) showing no evidence of hypermetabolic activity. An axial CT of the pelvis (F) showing the post-surgical empty space in the center of the pelvis occupied by the bowel with no evidence of malignancy. Abbreviations: T2W, T2-weighted; PET, positron emission tomography.

A CT of her abdomen/pelvis with contrast revealed a large right abdominal mass of 18.52 x 8.61 x 16.79 cm in size and another parallel heterogenous mass of 12.75 x 7.06 x 15.71 cm with extension into the pelvic region. An MRI of her abdomen/pelvis with contrast was performed for better visualization. The left-side mass was confirmed to be a leiomyoma, but the right mass had signal intensity properties different from a typical uterine leiomyoma, and therefore there were concerns for a malignancy. The differential diagnosis at this point included primary ovarian tumor vs. sarcoma vs. lymphoma (Figure 1).

Exploratory laparotomy with total abdominal hysterectomy (TAH)/bilateral salpingo-oophorectomy (BSO) was performed with the excision of draining lymph nodes; specimens were sent for histopathology.

**Figure 2:**
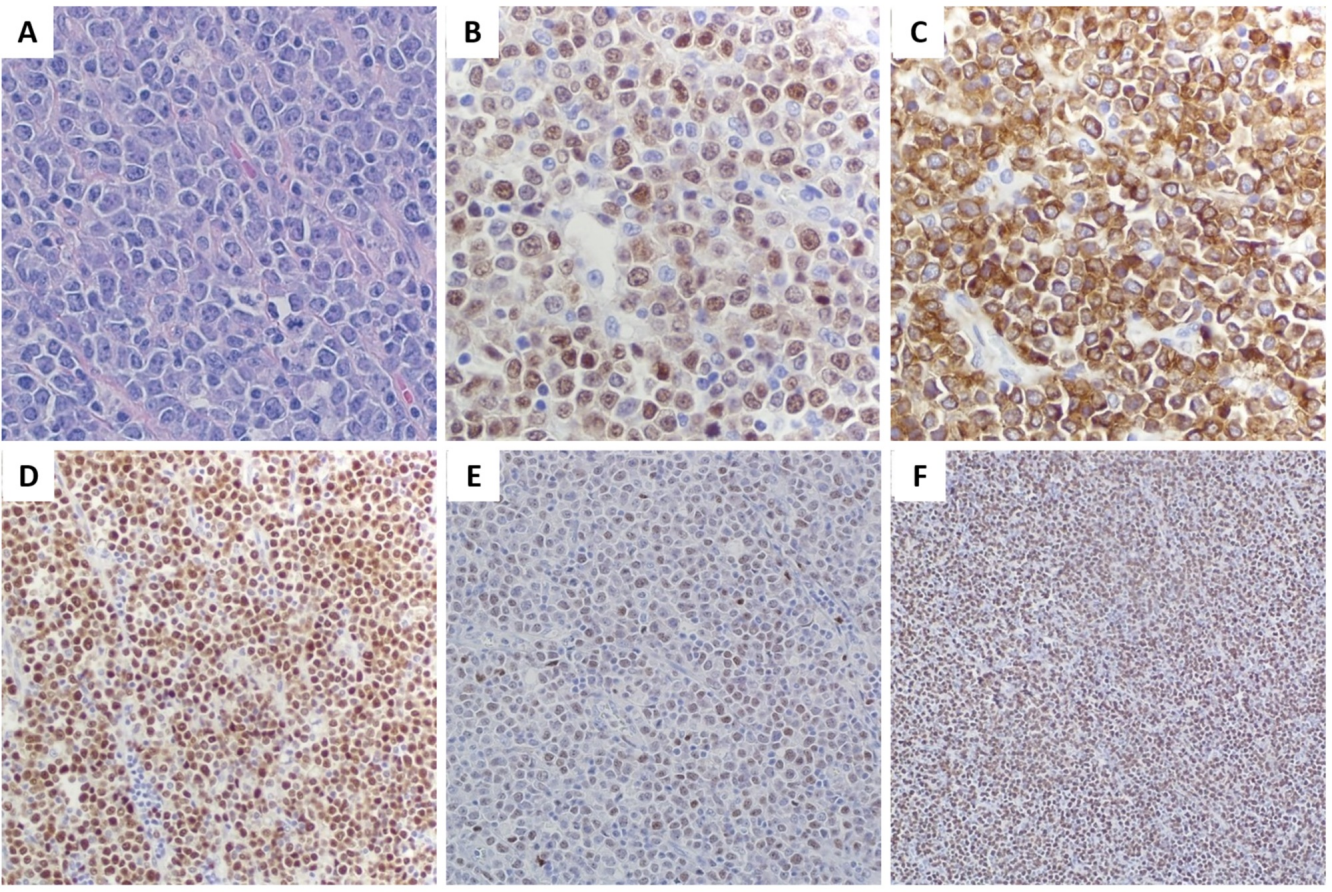
Histological and immunohistochemical findings of the surgically resected mass. (A) Hematoxylin-eosin staining (40X magnification) showing plasmablastic cells with prominent nucleoli. (B) Immunohistochemical staining is positive for MUM1, (C) CD79a, (D) PAX5, (E) BCL6. (F) The proliferation rate is 92% as represented by Ki-67 expression.

The histopathology report revealed neoplastic cells that completely effaced the underlying architecture of the right ovary, fallopian tubes, and lymph nodes. They were composed of monotonous large cells with a Ki-67 proliferative index of 92%, high nuclear/cytoplasmic ratio (N/C), high mitotic figures, amphophilic cytoplasm, eccentric nuclei with prominent nucleoli, and vesicular chromatin. Immunohistochemical staining revealed that tumor cells were positive for MUM1, CD79a, PAX5, BCL6, focally positive for BCL2 while showing negativity for CD20, CD10, CD30, CD138, CD45, CD3, CK AE1/AE3, inhibin, EMA, anaplastic lymphoma kinase (ALK), and negative connective tissue markers (relevant histological and immunohistochemical findings have been shown in Figure 2).

As the tumor cells were showing immunophenotypic similarities to plasma cell neoplasms, a workup for multiple myeloma, including serum protein electrophoresis (SPEP) with immunofixation was performed but was found to be negative.

The fluorescent in-situ hybridization (FISH) was positive for BCL6 gene rearrangement and negative for Epstein-Barr virus (EBV) RNA, BCL2, MYC, and CCND1/IGH rearrangements. Any metastasis to her brain and bone marrow was ruled out by flow cytometry analysis of cerebrospinal fluid (CSF) and bone marrow aspiration biopsy, respectively. Since these lymphomas have a strong association with chronic viral infections, HIV and HHV8 were ruled out using serological screening tests. Based on the morphologic and immunohistochemical profile, and extranodal clinical presentation, a diagnosis of plasmablastic lymphoma (PBL), a sub-type of CD20-negative DLBCL, was established.

An aggressive six cycles of chemotherapy with EPOCH regimen including etoposide, prednisolone, oncovin/vincristine, cyclophosphamide, and hydroxydaunorubicin with pre-tests for medication side effect profile was started. Furthermore, intrathecal methotrexate was added to the regimen for central nervous system (CNS) prophylaxis. The patient tolerated the treatment without any significant side-effects and responded well. The response to treatment was validated with a post-chemotherapy positron emission tomography (PET)-CT scan (Figures 1E, 1F). The patient is being monitored with a PET-CT pan-scan every six months and is currently in remission for the past year.

## Discussion

CD20-negative DLBCL constitutes 1-2% of all DLBCLs and is characterized by complex histology, genetics, and a high therapeutic resistance [8]. The most common subtype of CD20-negative DLBCL is PBL, constituting 75% of the total cases [2]. PBLs are predominant in men with an age of onset in the fourth decade of life. PBL has a strong propensity to involve extranodal sites like the oral cavity, gastrointestinal tract, and skin. The frequency of oral involvement is higher in HIV-positive (58%) than in HIV-negative patients (16%). Some of the less common sites include the central nervous system, paranasal sinus, mediastinum, lungs, liver, testes, retroperitoneum, parotid gland, and soft tissues. Bone marrow involvement has been reported in 30% of cases [9]. To the best of our review of the databases, the involvement of pelvic organs by PBL as seen in our case represents the only reported experience so far.

CD20 is a glycosylated phosphoprotein, expressed on the surface of all B cells except early pro-B cells and plasma cells. It is encoded by the MS4A1 gene located on chromosome 11q12.2. CD20 plays a pivotal role in the maturation, differentiation, and activation of B cells. Genetic mutations of MS4A1 lead to conformational changes in the protein and are believed to be one of the molecular mechanisms responsible for the CD20 negative phenotype [10,11]. Almost all B-cell NHLs are CD20-positive (except 1-2%) [1]. CD20 negativity imparts some characteristic features to these lymphomas like extranodal involvement, a more aggressive clinical course, as well as a lack of responsiveness to rituximab and conventional chemotherapy, thereby posing a unique challenge on the diagnostic and therapeutic forefront [12].

As PBL is frequently seen in patients with chronic HIV and/or EBV co-infection, viral serologies must be included in the diagnostic workup [13]. Our patient, however, had a negative HIV/EBV status. PBLs have also been reported in association with immunocompromised states like high-dose steroid therapy for autoimmune disorders/solid organ transplantation, pre-existing lymphoproliferative diseases, and advanced age [9].

On histopathology, PBL cells exhibit characteristics of a typical high-grade tumor. PBLs pose a diagnostic dilemma because they typically lack the expression of B-cell markers (CD19, CD20, PAX5) and show little-to-no expression of leukocyte common antigen CD45. Their immunophenotype resembles that of plasma cell neoplasms as they frequently express CD79a, IRF4/MUM1, BLIMP-1, CD38, and CD138, thus adding multiple myeloma (MM) and B-cell lymphoma, unclassifiable (BCUL) to the list of differentials [14]. PRDM1/BLIMP1 expression, in conjunction with the absence/low expression of CD20, can be used as a diagnostic marker for PBL identification [15]. The proliferation rate of PBL is generally high, with Ki-67 expression higher than 60%. The overall prognosis remains poor with various systematic reviews reporting median overall survival (OS) times ranging between 9 and 15 months [16].

Molecular analysis using in-situ hybridization techniques has an important prognostic value in PBL cases. Key molecular markers include the presence of MYC gene rearrangements and EBV-encoded RNA (EBER). MYC gene rearrangements can be seen in approximately 40% of cases of HIV-positive PBL and translate into a poor prognosis. EBER expression has been reported in approximately 80% of cases with HIV-positive PBL and in 50% of cases with HIV-negative PBL. This points towards a probable role of EBV in the pathogenesis of the disease [17]. Some of the good prognostic factors of PBL include Ann Arbor classification Stage 1, expression of EBER, CD45, and lack of c-Myc aberrations [16].

To date, there is no standard treatment regimen for PBL. Current guidelines recommend against the use of CHOP (cyclophosphamide, doxorubicin, vincristine, and prednisone) due to poor outcomes. Retrospective studies show favorable outcomes with the following regimens: CODOX-M/IVAC (cyclophosphamide, vincristine, doxorubicin, methotrexate alternating with ifosfamide, etoposide, cytarabine), dose adjusted EPOCH (infusion etoposide, vincristine and doxorubicin along with bolus cyclophosphamide and prednisone), and hyper-CVAD (cyclophosphamide, vincristine, doxorubicin and dexamethasone alternating with high-dose methotrexate and cytarabine) [9]. In addition to these regimens, intrathecal agents like methotrexate can also be included to minimize the risk of CNS involvement. Cases with concomitant HIV infections require initiation or optimization of highly active antiretroviral therapy by an infectious disease specialist. Recent reviews have shown that high-dose chemotherapy followed by autologous stem cell support (ASCT) could be a viable option in patients with PBL with the first occurrence. Interestingly, due to the plasmacytic nature of PBL, drugs from the myeloma line like bortezomib have been tried albeit with limited success. Case reports advocating the use of a combination of bortezomib and infusional dose-adjusted EPOCH, immunomodulatory drug lenalidomide, and brentuximab vedotin (in a patient with CD30-expressing relapsed PBL), are also present [9].

## Conclusions

In this report, we presented a rare case of CD20-negative DLBCL (PBL type) successfully treated with EPOCH. These are rare, aggressive lymphomas which pose diagnostic and therapeutic challenges. Differentials include plasma cell neoplasms, sarcomas, and melanomas. Patients typically have extranodal involvement, a more aggressive clinical course, and lack responsiveness to rituximab and conventional chemotherapy. Less conventional treatment modalities like ASCT, bortezomib, lenalidomide, and brentuximab have been tried with limited success. Given the rarity associated with this condition, large prospective studies are unlikely to be performed. However, their inclusion in larger clinical trials for aggressive B-cell lymphomas may yield promising results.
